# Synthesis, crystal structure and Hirshfeld surface analysis of the two-dimensional hydrogen-bonded network [TCNQ-H_2_]^2+^[AsF_6_]_2_^−^

**DOI:** 10.1107/S2056989025007108

**Published:** 2025-08-15

**Authors:** Malte Sellin, Susanne Margot Rupf, Moritz Malischewski

**Affiliations:** a Freie Universität Berlin Institut für Chemie und Biochemie - Anorganische Chemie Fabeckstrasse 34-36 14195 Berlin Germany; Universidad de Los Andes Mérida, Venezuela

**Keywords:** crystal structure, tetra­cyano­quinodi­methane, superacids, hydrogen bonded networks, nitriles

## Abstract

The structure of 2-[4-(di­cyano­meth­yl)cyclo­hexa-2,5-dien-1-yl]propane­bis­(nitrilium) bis­(hexa­fluorido­arsenate), C_12_H_6_N_4_^2+^·2AsF_6_^−^, has ortho­rhom­bic (*Cmce*) symmetry. The compound exhibits a layer structure, which is formed by hydrogen bonds between the semi-protonated nitrile groups. Unexpectedly, no H⋯F contacts are observed. Instead, the [AsF_6_]^−^ anions show C⋯F contacts to the positively polarized carbon atoms of the dication with distances in the range 2.871 (2)–3.154 (2) Å.

## Abstract 2

Tetra­cyano­quinodi­methane (TCNQ - C_12_H_4_N_4_) can be doubly protonated by the superacid HF/AsF_5_ to give crystals of [C_12_H_6_N_4_]^2+^[AsF_6_]^−^_2_.

## 1. Chemical context

Tetra­cyano­quinodi­methane (TCNQ) is widely used in organic semiconductors and in charge-transfer components (Torrance, 1979 ▸; Jérome, 2004 ▸; Phan *et al.*, 2015 ▸; Potember *et al.*, 1979 ▸). The weakly oxidizing properties and the stability of its radical anion and diamagnetic dianion have led to a large number of structurally characterized [TCNQ]^–·^ and [TCNQ]^2–^ salts (Singh *et al.*, 2016 ▸). The oxidation potential of TCNQ can be dramatically increased to *ca*. 0.9 V *vs* Fc^+/0^ by the coordination of the Lewis acid B(C_6_F_5_)_3_ to every nitrile group (Albrecht *et al.*, 2022 ▸). Although the treatment of those nitriles with such electrophiles forming Lewis acid–base adducts is a common approach, the protonation of nitriles requires superacids such as HF/*E*F_5_ (*E* = As, Sb) or HSO_3_F/SbF_5_ (Olah & Kiovsky, 1968 ▸). A few years ago, the crystal structures of some protonated nitriles were reported, including *e.g.* [H_3_CCN-H]^+^[AsF_6_]^−^ and [H_5_C_6_CN-H]^+^[AsF_6_]^−^ (Haiges *et al.*, 2016 ▸). Protonations of cyano­metalates are on the other hand much more common due to the stronger basicity coming from the negative charge of the complex. Superacids can be used to convert octa­cyano­metalates to their respective homoleptic hydrogen isocyanide complexes [*M*(CNH)_8_]^4+^([SbF_6_]^−^)_4_*(M* = Mo, W; Sellin *et al.*, 2020 ▸).
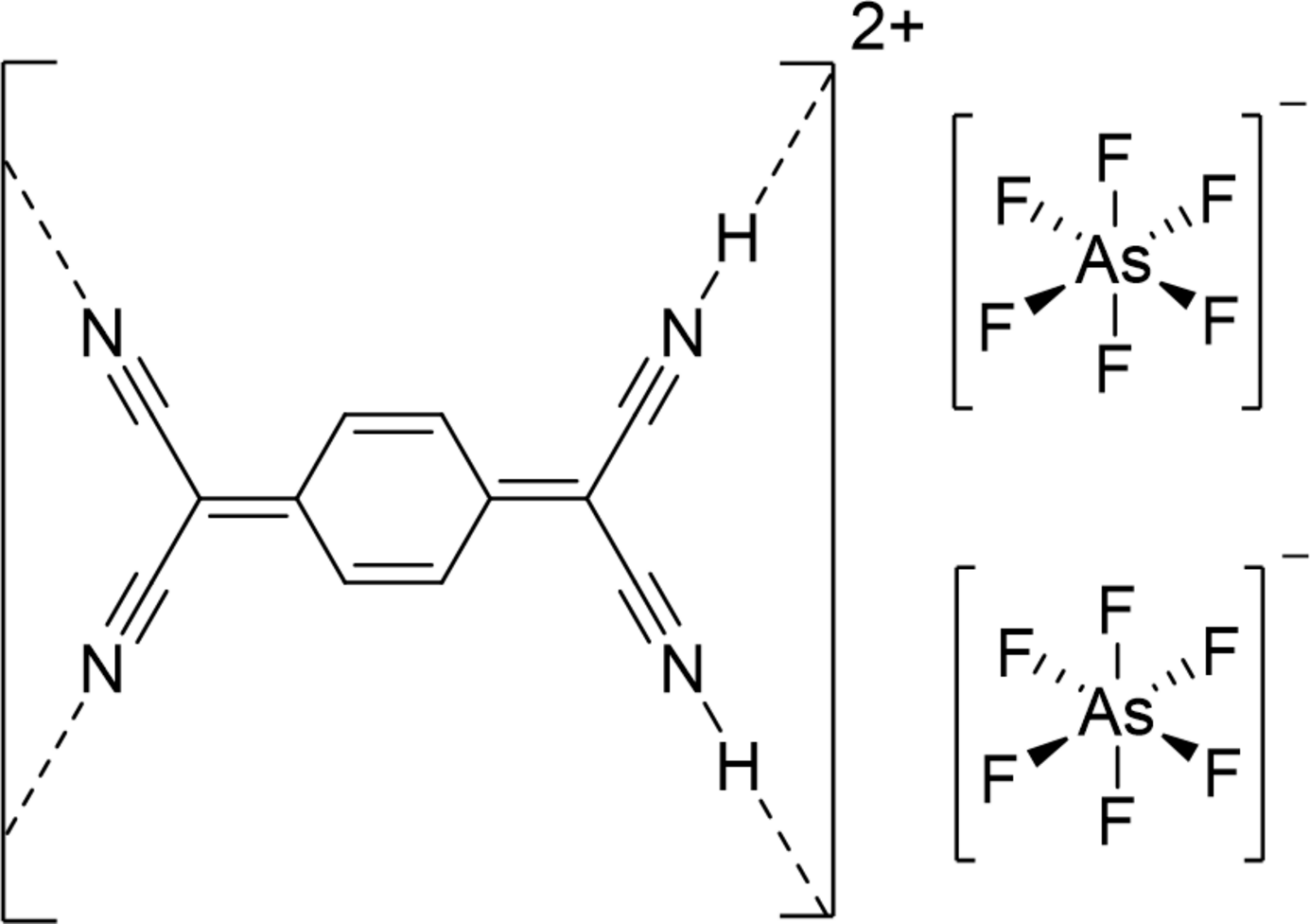


In this work we investigated the reactivity of TCNQ with HF/AsF_5_. Instead of the expected fourfold protonation, we observed a di-periodic layered hydrogen-bonded network between diprotonated TCNQ moieties. In the following, its solid-state structure will be discussed.

## 2. Structural commentary

**Figure 1:**
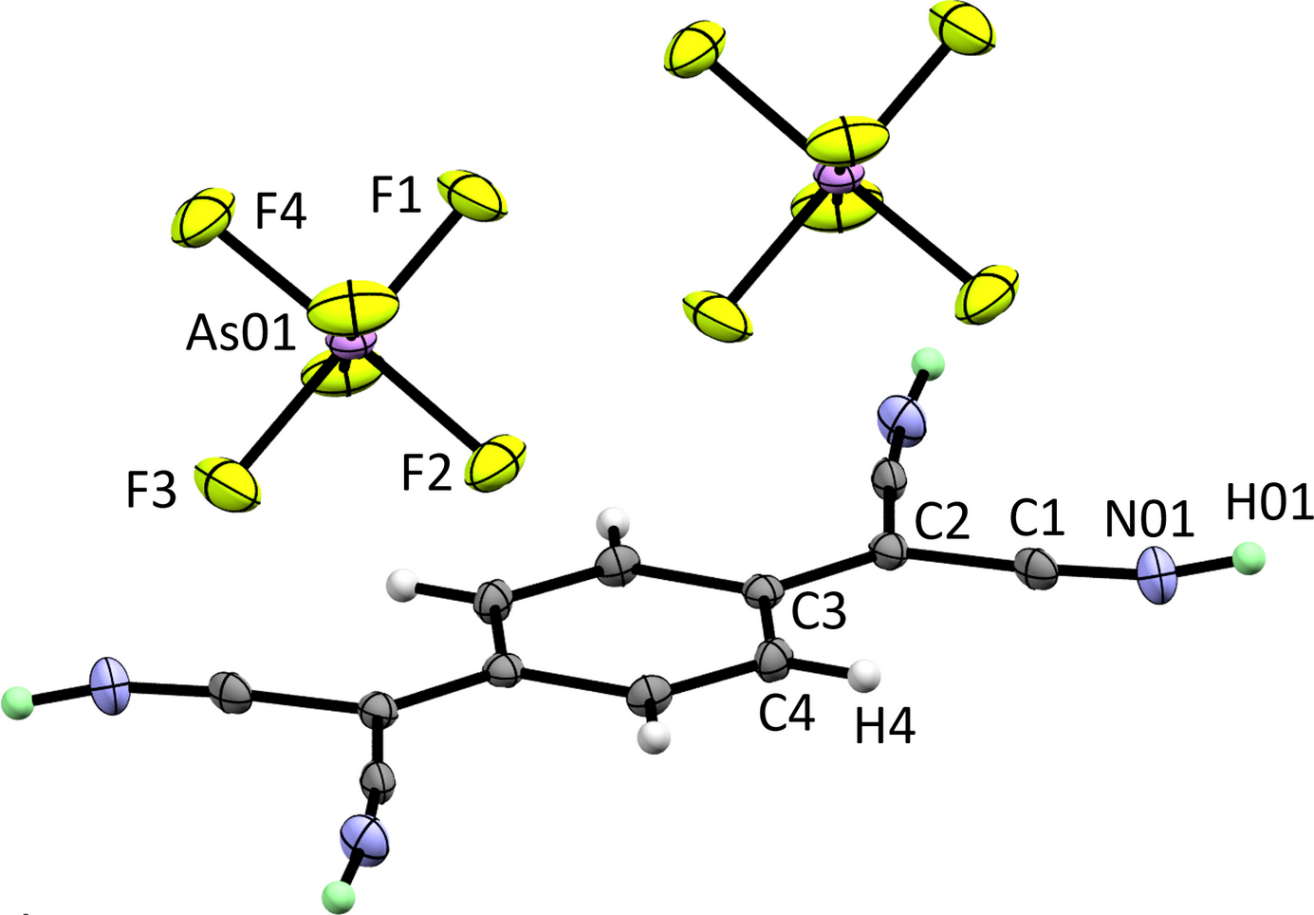
The mol­ecular structure of the title compound. Displacement ellipsoids shown at the 50% probability level. Hydrogen atoms displayed in mint have an site-occupancy factor (SOF) of 0.5.

**Figure 2:**
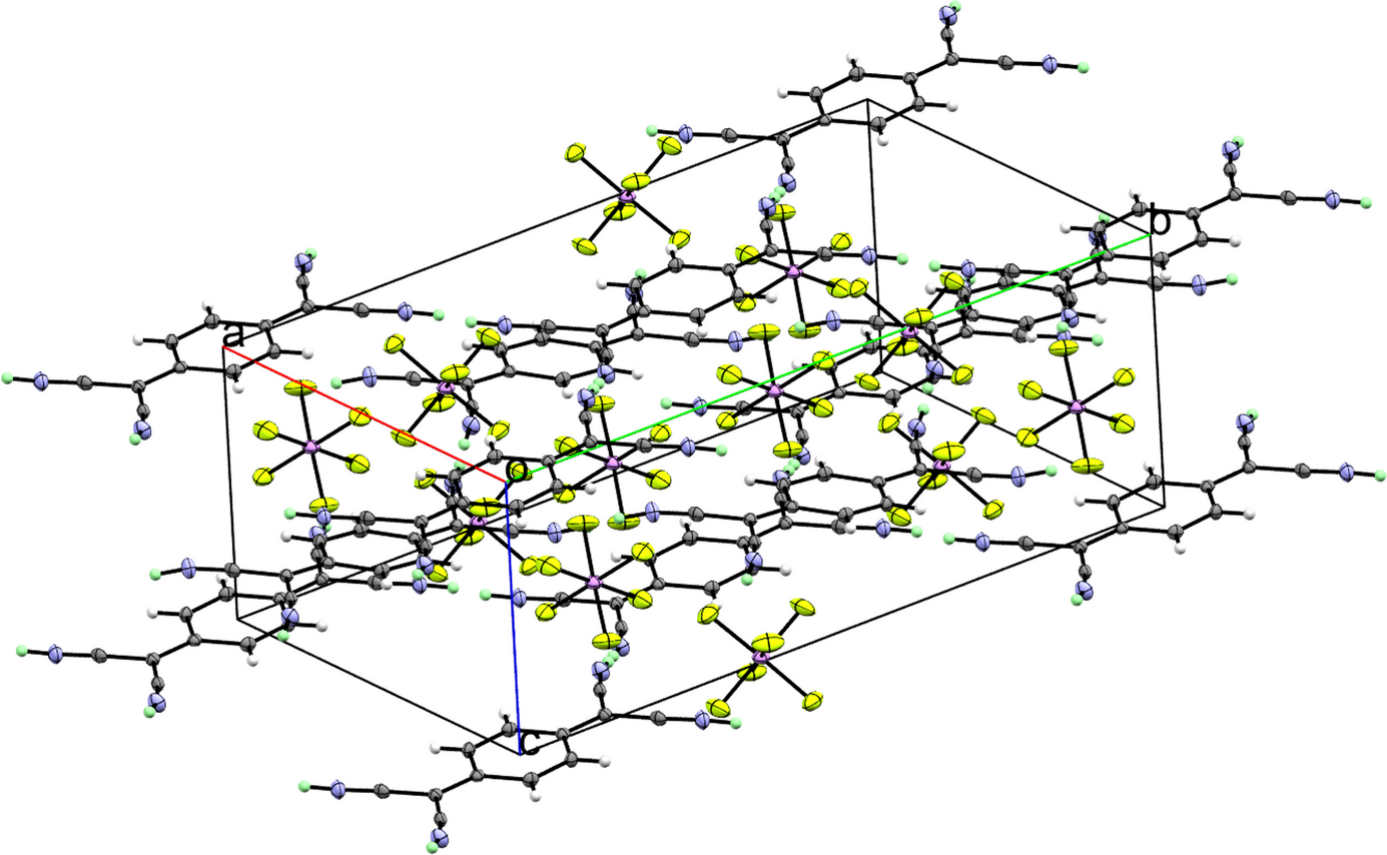
Packing of the title compound in the unit cell. Displacement ellipsoids shown at the 50% probability level. Colour code: arsenic – purple; fluorine – light green; nitro­gen – blue; carbon – dark grey; ordered hydrogen – white; hydrogen in PART −1 – mint.

[TCNQ-H_2_]^2+^[AsF_6_]^−^_2_ ▸ crystallizes in the ortho­rhom­bic space group *Cmce*. The packing is best described as a distorted NaCl structure with a close-packed [TCNQ-H_2_]^2+^ network and one ([AsF_6_]^−^)_2_ moiety in each distorted octa­hedral void (Fig. 2 ▸). The asymmetric unit consists of a {CH–C_2_–CNH} and an {AsF_4_} unit (Fig. 1 ▸), whereas H01 is disordered over the mirror plane (−*x*, *y*, *z*) with a SOF of 0.5 giving the overall formula C_12_H_6_N_4_As_2_F_12_. The inter­molecular distances between the nitrile groups are 2.542 (2) Å. Upon coordination of electrophiles to a C—N group, contraction of the C—N bond is expected due to electrostatic effects. Comparison to the structure of non-protonated TCNQ (Krause *et al.*, 2015 ▸) reveals that the C—N bond of TCNQ shortens by 0.02 Å to 1.130 (2) Å upon semi-protonation. The six-membered ring in the dication is clearly identified as a quinoidal system [bond lengths C3—C4: 1.450 (2) and C4—C4′: 1.342 (3) Å].

## 3. Supra­molecular features

**Figure 3:**
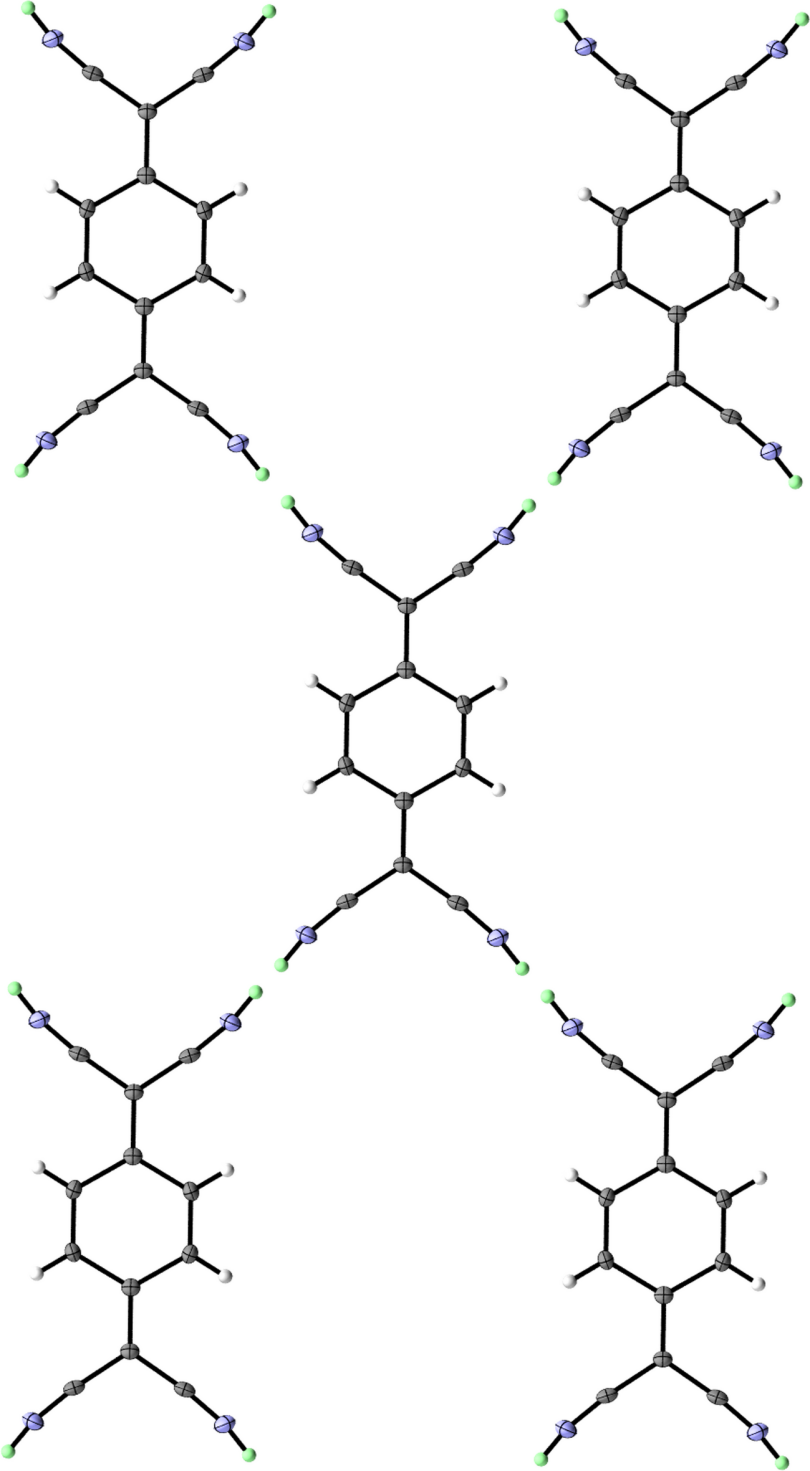
The di-periodic hydrogen-bonded network of the diprotonated TCNQ moieties. Displacement ellipsoids are shown at the 50% probability level. Colour code: arsenic – purple; fluorine – light green; nitro­gen – blue; carbon dark grey; ordered hydrogen – white; hydrogen in PART −1 – mint.

**Figure 4:**
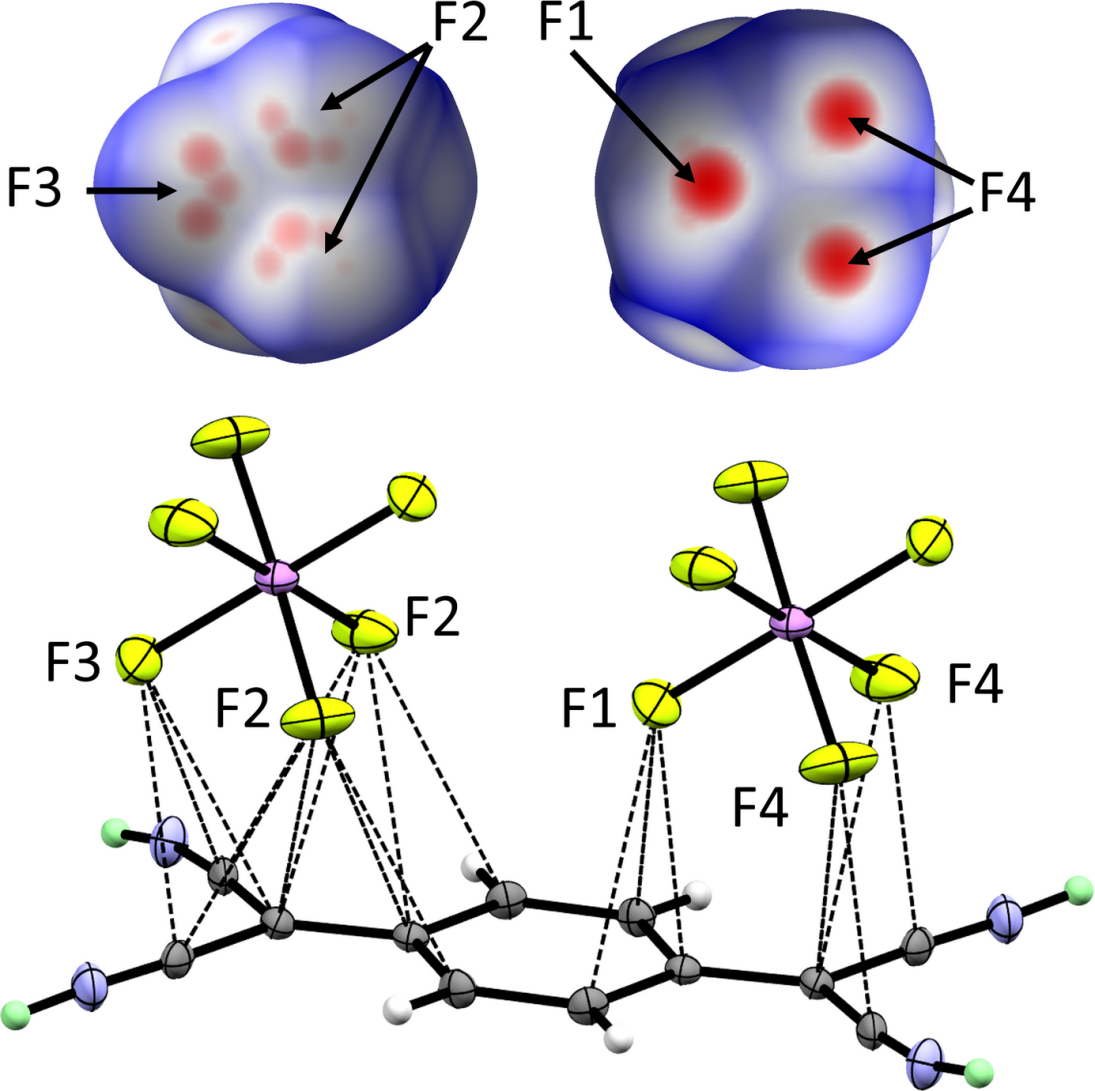
Top: Hirshfeld surface of the [AsF_6_]^−^ anion. Colour ranges from red (short contacts) over white to blue (long contacts). Bottom: Contacts of the [AsF_6_]^−^ anions with the π-system of the diprotonated TCNQ. Displacement ellipsoids shown at the 50% probability level. Colour code: arsenic – purple; fluorine – light green; nitro­gen – blue; carbon dark grey; ordered hydrogen – white; disordered hydrogen in PART −1 – mint.

In the solid-state structures of the literature-known protonated nitriles, CN—H⋯F contacts are the dominant motif regarding cation–anion inter­actions (Haiges *et al.*, 2016 ▸). However, in this structure, this motif is not observed. Instead, strong, symmetric CN—H⋯NC hydrogen bonds are formed (Table 1 ▸, Fig. 3 ▸). However, the complete absence of H⋯F contacts in a crystal structure of a protonated nitrile is very surprising. Instead, the [AsF_6_]^−^ anions are located directly over and under the electron-deficient π-system of the [TCNQ-H_2_]^2+^ moiety (Fig. 4 ▸). Three fluorine atoms of the [AsF_6_]^−^ anion point directly to the electron deficient carbon atoms, leading to three stronger C⋯F contacts [F1—As01—C2—C3 torsion angle = 0.0 (1)°]. The other three fluorine atoms are found in a staggered geometry towards the dication [F2—As01—C2—C3 torsion angle = 61.6 (1)°], allowing more, but weaker, C⋯F contacts (Table 2 ▸ ▸). These inter­molecular C⋯F contacts can also be visualized with a Hirshfeld surface (Hirshfeld *et al.*, 1977 ▸) in *CrystalExplorer* (Turner *et al.*, 2017 ▸). This surface shows at the F1 and F4 sites three strong inter­actions (red) and, at the other sites, multiple smaller inter­actions for F2 and F3 (pink). Still, the C⋯F contacts are rather long in the present structure (shortest C⋯F contact = 2.894 Å). A salt of triprotonated 1,3,5-tri­cyano­benzene [C_6_(CNH)_3_H_3_]^3+^[Sb_2_F_11_]^−^_2_[SbF_6_]^−^ (Nitzer *et al.*, 2022*a* ▸) has C⋯F contacts in the same range, as does triprotonated 1,3,5-tri­carb­oxy­benzene [C_6_(CO_2_H_2_)_3_H_3_]^3+^[SbF_6_]^−^_3_ (Nitzer *et al.*, 2022*b* ▸). Besides the protonated aromatic compounds, de-electronated/oxidized aromatic compounds also have similarly short C⋯F contacts, *e.g.* the hexa­fluoro­benzene radical–cation salt [C_6_F_6_]^+^[Sb_2_F_11_]^−^ (Shorafa *et al.*, 2009 ▸), the hexa­methyl­benzene dication (Malischewski *et al.*, 2017*a* ▸,*b* ▸) and the cyclo­penta­dienium radical–cation salt [C_5_(C_6_F_5_)_5_]^+^[Sb_3_F_16_]^−^ (Schulte *et al.*, 2024 ▸) and related perhalogenated dimers (Rupf *et al.*, 2020 ▸).

## 4. Database survey

A survey of the CSD (version 5.46, update June 2024; Groom *et al.*, 2016 ▸) gave 1451 hits including the TCNQ moiety. While many charge-transfer salts are known, the poor basicity of the TCNQ leads to a rare role as Lewis base with only 235 hits in the CSD and 226 of them metals. In contrast, only five non-metal coordinations are known [all to B(C_6_F_5_)_3_]. While the neutral TCNQ moiety coordinates only two B(C_6_F_5_)_3_, the [TCNQ]^-/2-^ coordinates B(C_6_F_5_)_3_ on all four nitrile functions. There are 74 hits for the –CN—H motif, but only 15 of them refer to protonated nitriles (C—CN—H).

## 5. Synthesis and crystallization

1.0 mL of anhydrous hydrogen fluoride, 1.0 mL of sulfur dioxide and arsenic penta­fluoride (64 mg, 0.4 mmol, 4.0 eq.) were condensed on TCNQ (20 mg, 0.1 mmol, 1.0 eq.) at 77 K. The solution was slowly warmed to room temperature leading to a clear orange solution. The solution was then slowly cooled down to 195 K over the course of a few days to afford yellow crystalline blocks suitable for single crystal X-ray diffraction analysis in *ca*. 80% yield.

## 6. Refinement

Crystal data, data collection and structure refinement details are summarized in Table 3 ▸.

## Supplementary Material

Crystal structure: contains datablock(s) I. DOI: 10.1107/S2056989025007108/dj2080sup1.cif

Structure factors: contains datablock(s) I. DOI: 10.1107/S2056989025007108/dj2080Isup2.hkl

CCDC reference: 2478779

Additional supporting information:  crystallographic information; 3D view; checkCIF report

## Figures and Tables

**Table 1:** Hydrogen-bond geometry (Å, °)

*D*—H⋯*A*	*D*—H	H⋯*A*	*D*⋯*A*	*D*—H⋯*A*
N01—H01⋯N01^i^	0.86 (4)	1.69 (4)	2.544 (2)	177 (7)

Symmetry code: (i) 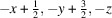.

**Table 2:** F⋯C contacts between the [AsF_6_]^−^ counter-ions and the [TCNQ-H_2_]^2+^ moieties (Å)

site 1 (F1+F4)		site 2 (F2+F3)			
C1⋯F4	2.893 (2)	C1⋯F2	3.097 (2)	C1⋯F3	3.053 (2)
C2⋯F4	3.152 (2)	C2⋯F3	3.091 (3)	C2⋯F2	3.064 (2)
C3⋯F1	2.869 (3)	C3⋯F2	3.115 (2)		
C4⋯F1	3.124 (2)	C4⋯F2	3.155 (2)		

**Table 3:** Experimental details

Crystal data	
Chemical formula	C_12_H_6_N_4_^2+^·2AsF_6_^−^
*M*_r_	584.05
Crystal system, space group	Orthorhombic, *Cmce*
Temperature (K)	100
*a*, *b*, *c* (Å)	11.3963 (4), 20.9925 (9), 7.6198 (3)
*V* (Å^3^)	1822.94 (12)
*Z*	4
Radiation type	Mo *K*α
μ (mm^−1^)	3.79
Crystal size (mm)	0.36 × 0.18 × 0.11

Data collection	
Diffractometer	Bruker APEXII CCD
Absorption correction	Multi-scan (*SADABS*; Krause et al., 2015 ▸)
*T*_min_, *T*_max_	0.635, 0.745
No. of measured, independent and observed [*I* > 2σ(*I*)] reflections	3524, 985, 976
*R*_int_	0.013
(sin θ/λ)_max_ (Å^−1^)	0.626

Refinement	
*R*[*F*^2^ > 2σ(*F*^2^)], *wR*(*F*^2^), *S*	0.019, 0.047, 1.12
No. of reflections	985
No. of parameters	84
H-atom treatment	All H-atom parameters refined
Δρ_max_, Δρ_min_ (e Å^−3^)	0.40, −0.43

Computer programs: *APEX3* and *SAINT* (Bruker, 2018 ▸), *SHELXT2014/5* (Sheldrick, 2015*a* ▸), *SHELXL* (Sheldrick, 2015*b* ▸) and *OLEX2*Dolomanov *et al.*, 2009 ▸).

## supplementary crystallographic information

### 2-[4-(Dicyanomethyl)cyclohexa-2,5-dien-1-yl]propanebis(nitrilium) bis(hexafluoridoarsenate) Crystal data

**Table:**

C_12_H_6_N_4_^2+^·2AsF_6_^−^	*D*_x_ = 2.128 Mg m^−^^3^
*M**_r_* = 584.05	Mo *K*α radiation, λ = 0.71073 Å
Orthorhombic, *C**m**c**e*	Cell parameters from 9847 reflections
*a* = 11.3963 (4) Å	θ = 3.3–26.4°
*b* = 20.9925 (9) Å	µ = 3.79 mm^−^^1^
*c* = 7.6198 (3) Å	*T* = 100 K
*V* = 1822.94 (12) Å^3^	Block, yellow
*Z* = 4	0.36 × 0.18 × 0.11 mm
*F*(000) = 1120	

### 2-[4-(Dicyanomethyl)cyclohexa-2,5-dien-1-yl]propanebis(nitrilium) bis(hexafluoridoarsenate) Data collection

**Table:**

Bruker APEXII CCD diffractometer	976 reflections with *I* > 2σ(*I*)
φ and ω scans	*R*_int_ = 0.013
Absorption correction: multi-scan (*SADABS-2016/1*; Krause et al., 2015)	θ_max_ = 26.4°, θ_min_ = 3.3°
*T*_min_ = 0.635, *T*_max_ = 0.745	*h* = −12→14
3524 measured reflections	*k* = −26→12
985 independent reflections	*l* = −9→9

### 2-[4-(Dicyanomethyl)cyclohexa-2,5-dien-1-yl]propanebis(nitrilium) bis(hexafluoridoarsenate) Refinement

**Table:**

Refinement on *F*^2^	Primary atom site location: structure-invariant direct methods
Least-squares matrix: full	Hydrogen site location: difference Fourier map
*R*[*F*^2^ > 2σ(*F*^2^)] = 0.019	All H-atom parameters refined
*wR*(*F*^2^) = 0.047	*w* = 1/[σ^2^(*F*_o_^2^) + (0.0178*P*)^2^ + 4.242*P*] where *P* = (*F*_o_^2^ + 2*F*_c_^2^)/3
*S* = 1.12	(Δ/σ)_max_ = 0.001
985 reflections	Δρ_max_ = 0.40 e Å^−^^3^
84 parameters	Δρ_min_ = −0.43 e Å^−^^3^
0 restraints	

### 2-[4-(Dicyanomethyl)cyclohexa-2,5-dien-1-yl]propanebis(nitrilium) bis(hexafluoridoarsenate) Special details

**Table:**

Geometry. All esds (except the esd in the dihedral angle between two l.s. planes) are estimated using the full covariance matrix. The cell esds are taken into account individually in the estimation of esds in distances, angles and torsion angles; correlations between esds in cell parameters are only used when they are defined by crystal symmetry. An approximate (isotropic) treatment of cell esds is used for estimating esds involving l.s. planes.
Refinement. Hydrogen atoms at the nitrogen nuclei were refined in PART -1

### 2-[4-(Dicyanomethyl)cyclohexa-2,5-dien-1-yl]propanebis(nitrilium) bis(hexafluoridoarsenate) Fractional atomic coordinates and isotropic or equivalent isotropic displacement parameters (Å^2^)

**Table:**

	*x*	*y*	*z*	*U*_iso_*/*U*_eq_	Occ. (<1)
As01	0.500000	0.62594 (2)	0.48213 (3)	0.01821 (10)	
F2	0.60728 (10)	0.59600 (6)	0.61714 (15)	0.0336 (3)	
F1	0.500000	0.55815 (8)	0.3601 (2)	0.0402 (5)	
F4	0.39438 (12)	0.65848 (7)	0.34763 (15)	0.0399 (3)	
F3	0.500000	0.69499 (8)	0.6063 (2)	0.0392 (5)	
C2	0.500000	0.63170 (11)	−0.0275 (3)	0.0133 (5)	
C3	0.500000	0.56661 (11)	−0.0157 (3)	0.0130 (4)	
C4	0.39021 (14)	0.53182 (8)	−0.0085 (2)	0.0147 (3)	
H4	0.3230 (18)	0.5540 (9)	−0.014 (2)	0.014 (5)*	
C1	0.39634 (15)	0.66974 (8)	−0.0308 (2)	0.0153 (3)	
N01	0.32043 (14)	0.70440 (7)	−0.0314 (2)	0.0212 (3)	
H01	0.274 (4)	0.7358 (18)	−0.014 (6)	0.017 (11)*	0.5

### 2-[4-(Dicyanomethyl)cyclohexa-2,5-dien-1-yl]propanebis(nitrilium) bis(hexafluoridoarsenate) Atomic displacement parameters (Å^2^)

**Table:**

	*U*^11^	*U*^22^	*U*^33^	*U*^12^	*U*^13^	*U*^23^
As01	0.02267 (16)	0.02017 (15)	0.01179 (14)	0.000	0.000	0.00001 (9)
F2	0.0290 (6)	0.0535 (8)	0.0182 (5)	0.0138 (6)	−0.0012 (5)	0.0026 (5)
F1	0.0777 (14)	0.0240 (8)	0.0189 (8)	0.000	0.000	−0.0067 (7)
F4	0.0408 (7)	0.0594 (9)	0.0194 (6)	0.0183 (6)	−0.0044 (5)	0.0050 (6)
F3	0.0748 (14)	0.0217 (8)	0.0211 (8)	0.000	0.000	−0.0023 (7)
C2	0.0162 (11)	0.0125 (11)	0.0113 (10)	0.000	0.000	−0.0006 (8)
C3	0.0159 (11)	0.0139 (11)	0.0092 (10)	0.000	0.000	−0.0006 (8)
C4	0.0115 (8)	0.0166 (8)	0.0160 (8)	0.0022 (7)	0.0000 (6)	0.0002 (6)
C1	0.0208 (9)	0.0103 (7)	0.0150 (8)	−0.0040 (7)	0.0005 (6)	0.0001 (6)
N01	0.0226 (8)	0.0144 (7)	0.0267 (8)	0.0042 (7)	0.0015 (6)	0.0005 (6)

### 2-[4-(Dicyanomethyl)cyclohexa-2,5-dien-1-yl]propanebis(nitrilium) bis(hexafluoridoarsenate) Geometric parameters (Å, º)

**Table:**

As01—F2	1.7170 (11)	C2—C1	1.426 (2)
As01—F2^i^	1.7170 (11)	C3—C4^i^	1.450 (2)
As01—F1	1.6999 (16)	C3—C4	1.450 (2)
As01—F4^i^	1.7221 (12)	C4—C4^ii^	1.342 (3)
As01—F4	1.7221 (12)	C4—H4	0.90 (2)
As01—F3	1.7312 (17)	C1—N01	1.130 (2)
C2—C3	1.369 (3)	N01—H01	0.86 (4)
C2—C1^i^	1.426 (2)		
			
F2^i^—As01—F2	90.80 (8)	F4^i^—As01—F3	89.61 (6)
F2^i^—As01—F4^i^	178.08 (7)	F4—As01—F3	89.61 (6)
F2—As01—F4^i^	90.23 (6)	C3—C2—C1^i^	124.05 (10)
F2^i^—As01—F4	90.24 (6)	C3—C2—C1	124.05 (10)
F2—As01—F4	178.08 (7)	C1^i^—C2—C1	111.9 (2)
F2^i^—As01—F3	88.79 (6)	C2—C3—C4	120.34 (11)
F2—As01—F3	88.80 (6)	C2—C3—C4^i^	120.34 (11)
F1—As01—F2	91.22 (6)	C4^i^—C3—C4	119.3 (2)
F1—As01—F2^i^	91.22 (6)	C3—C4—H4	118.3 (13)
F1—As01—F4^i^	90.37 (6)	C4^ii^—C4—C3	120.34 (11)
F1—As01—F4	90.37 (6)	C4^ii^—C4—H4	121.4 (13)
F1—As01—F3	179.98 (8)	N01—C1—C2	173.94 (18)
F4^i^—As01—F4	88.68 (9)	C1—N01—H01	166 (3)
			
C2—C3—C4—C4^ii^	−178.0 (2)	C1—C2—C3—C4	1.0 (3)
C4^i^—C3—C4—C4^ii^	1.4 (4)	C1^i^—C2—C3—C4	178.36 (18)
C1—C2—C3—C4^i^	−178.36 (18)	C1^i^—C2—C3—C4^i^	−1.0 (3)

Symmetry codes: (i) −*x*+1, *y*, *z*; (ii) *x*, −*y*+1, −*z*.

### 2-[4-(Dicyanomethyl)cyclohexa-2,5-dien-1-yl]propanebis(nitrilium) bis(hexafluoridoarsenate) Hydrogen-bond geometry (Å, º)

**Table:**

*D*—H···*A*	*D*—H	H···*A*	*D*···*A*	*D*—H···*A*
N01—H01···N01^iii^	0.86 (4)	1.69 (4)	2.544 (2)	177 (7)

Symmetry code: (iii) −*x*+1/2, −*y*+3/2, −*z*.
